# Regional Anesthesia Versus Systemic Analgesia in Thoracotomies for Congenital Cardiac Surgery: A Retrospective Case Series in a Quaternary Care Children's Hospital

**DOI:** 10.7759/cureus.107126

**Published:** 2026-04-15

**Authors:** Michael A Evans, Lav Patel, Sara Izzo, Santhanam Suresh, David J Krodel

**Affiliations:** 1 Department of Anesthesiology, Division of Pediatric Anesthesiology, Ann and Robert H. Lurie Children's Hospital of Chicago, Northwestern University Feinberg School of Medicine, Chicago, USA; 2 Department of Anesthesiology, Division of Pediatric Anesthesiology, University of Arizona College of Medicine, Phoenix, USA; 3 Department of Anesthesiology and Perioperative Medicine, Loyola University Chicago Stritch School of Medicine, Chicago, USA

**Keywords:** congenital cardiac surgery, neuraxial anesthesia, pediatric anesthesia, regional anesthesia, thoracotomy

## Abstract

Background/objectives

Limited studies have characterized differences in postoperative outcomes between regional anesthetic techniques with systemic analgesia and systemic analgesia alone in thoracotomies for congenital cardiac surgery. This retrospective case series aimed to assess the efficacy of regional anesthetic techniques in improving postoperative outcomes in children undergoing thoracotomies for cardiac surgery in comparison to children who only received systemic analgesia without any regional anesthesia.

Methods

Perioperative data from patients under 18 years of age who underwent thoracotomy for coarctation of the aorta or vascular ring repair between January 2017 and March 2022 in a single-center quaternary care children's hospital were collected, including the presence or absence of a regional or neuraxial anesthetic technique. Primary outcomes collected were 24-hour postoperative pain scores and 24-hour opioid consumption. Secondary outcomes collected were incidence of moderate pain, time to extubation, and block complications.

Results

One hundred and forty patients underwent thoracotomy for coarctation or vascular ring in the study period. Regional or neuraxial anesthesia (paravertebral, erector spinae plane, serratus anterior, or caudal opioid) was utilized in 62 (44.3%) cases. Paravertebral blocks were the most common block. Both groups demonstrated well-controlled pain throughout the first 24 hours. Twenty-four-hour opioid consumption (mg/kg of intravenous morphine equivalents (IME)) was similar between regional and non-regional groups (0.36 (0.20, 0.54) versus 0.39 (0.18, 0.62); p=0.48). There was no statistically significant difference in the incidence of moderate pain between groups.

Conclusions

Pain was well-controlled in patients who received regional plus systemic analgesia as well as patients who received systemic analgesia alone, with no differences in pain scores, moderate pain, or opioid use between the patient populations.

## Introduction

Congenital heart disease (CHD) affects nearly 40,000 (1%) of all births per year in the United States [1]. Congenital heart lesions, including pathologies like coarctation of the aorta and vascular rings, are frequently surgically addressed with thoracotomy. Given this surgical approach, adequate postoperative pain control is essential to promote patient comfort, improve respiratory status, and minimize stress-induced neuroendocrine responses [2]. Adequate postoperative pain management after cardiothoracic surgery is often challenging, particularly in the pediatric population. Many pediatric patients experience significant acute postoperative pain after thoracic surgery, with nearly 23% of them ultimately developing chronic pain [3]. While opioids have long been a cornerstone of postoperative pain control, many adverse events in the postoperative period are attributed to them [4]. Use of opioids increases the risk of respiratory complications, cardiovascular instability, prolonged intubation times, and opioid habituation in children [4,5]. Opioid-minimizing strategies, including multimodal analgesia, are increasingly being adopted in pediatric cardiothoracic surgery in hopes of reducing acute and chronic adverse events.

Regional anesthesia has emerged as a promising and safe method of postoperative pain control in children. There are several anatomical approaches for regional anesthesia and neuraxial anesthesia in CHD patients undergoing thoracotomy, including erector spinae, paravertebral, caudal, and serratus anterior plane blocks [6-10]. Neuraxial and regional anesthesia techniques have been demonstrated as safe in cardiac surgery, with evidence supporting earlier extubation, improved respiratory function, attenuated stress response, decreased pain scores, and reduced IV opioid requirements postoperatively [6,7]. A meta-analysis by Monahan et al. found that the addition of regional anesthesia techniques to systemic analgesia reduced postoperative pain for up to 24 hours in cardiac surgeries, with no difference in morbidity or mortality [11]. However, few studies have characterized differences in regional anesthesia techniques and systemic analgesia for pediatric cardiac surgery by way of thoracotomy, especially regarding postoperative opioid consumption, an important contributor to acute and chronic postoperative complications.

The objective of this study was to present a descriptive, observational comparison of our institutional practice patterns of routine regional anesthesia applications with systemic analgesia in children undergoing thoracotomy for cardiac surgery in comparison to children receiving only systemic analgesia. As a retrospective practice audit, it is therefore not intended as a comparative-effectiveness study. The primary outcomes of this study were pain scores in the first 24 hours post-block and total opioid consumption during the first 24 postoperative hours; secondary outcomes were the proportion of patients with an episode of moderate pain (at least one pain score ≥4/10 in the 24-hour postoperative period) [12], time to extubation, and regional block complications. 

We hypothesized that pain scores and opioid consumption in the first 24 hours postoperatively would be lower in the group which received both systemic analgesia and regional anesthesia.

## Materials and methods

Study design

A retrospective review of all pediatric cardiac surgical patients who underwent coarctation of the aorta or vascular ring repair at Ann and Robert H. Lurie Children's Hospital of Chicago, a single quaternary care pediatric hospital in Chicago, Illinois, between January 2017 and March 2022 was conducted. All patients under 18 years of age at the time of surgery who had a thoracotomy to approach their cardiac lesion (either coarctation of the aorta or vascular ring repair) were included in the study. Patients over the age of 18 years, patients who required cardiopulmonary bypass, and patients who had a separate surgical approach (such as sternotomy) in order to address their coarctation of the aorta or vascular ring repair were excluded. The Ann and Robert H. Lurie Children's Hospital of Chicago Institutional Review Board designated this study as exempt and waived the requirement for written informed consent (2022-5177; 01-25-2022). 

Patient demographic data, including age, sex, and American Society of Anesthesiologists (ASA) physical status/comorbidities, as well as perioperative variables of interest, such as medications, surgical and anesthesia times, extubation details, regional block specifics, postoperative pain scores, and surgical and anesthetic complications, were collected. Regional and neuraxial anesthetic techniques for this procedure included caudal epidural opioid, erector spinae plane, paravertebral, and serratus anterior plane blocks. Pain scores were documented by the bedside nurse, with pain scale type selected per institutional protocol (NIPS, rFLACC, FACES, NRS, Verbal), which were then normalized to a 0-10 scale for data analysis. This normalization was necessary as different nurses used different scales for the same patient and sometimes even different scales during the same shift for the same patient. The variation in pain scales used, therefore, prevented grouping patients by what pain scale was applied and forced standardization in this retrospective setting. Opioids administered in the first 24 hours were determined from the medication administration record and then converted to IV morphine equivalents.

As a retrospective audit, intraoperative management (including opioid and non-opioid adjuncts) and postoperative intensive care unit (ICU) protocols were not strictly standardized, especially over a five-year period. However, at baseline, patients received multimodal analgesic regimens perioperatively, at the discretion of the anesthesiologists and intensivists managing the patients. Postoperatively, patient analgesics and adjuncts were managed by the institutional pain service from the point of extubation onward. Given that intraoperative opioid selection and dosing were not standardized, intraoperative opioid dosing was therefore not included in the analysis. 

Statistical analysis

All baseline patient demographic and perioperative details were reported using mean and standard deviation or median and interquartile range for continuous variables. Frequencies and percentages were utilized for categorical variables. Minimum and maximum values were reported. Wilcoxon rank-sum tests, Pearson's chi-squared tests, and Fisher's exact tests were used to evaluate the unadjusted associations for each variable as well as regional anesthesia status, age group, and maximum pain score groups. A multivariate logistic regression model was generated to report the associations between perioperative characteristics (age group, regional anesthesia, surgery, and morphine consumption) and maximum pain score. Logistic regression results are reported as an odds ratio, with 95% confidence interval (CI) and p-values <0.05 being considered statistically significant.

All available data were included in this retrospective study. Given the retrospective study, there were no standard or prescribed assessment intervals for pain scores as would be present in a prospective study, so the number of pain assessments per patient in the first 24 hours varied as a result.

## Results

The final cohort consisted of 140 unique patients who met the eligibility criteria. Demographic data for all patients who received thoracotomy for coarctation of the aorta or vascular ring during the study period can be found in Table 1.

**Table 1:** Demographic data for all patients who received thoracotomy during the study period (age groups and regional anesthesia status) ^1^n (%)

	<6 months		6 months to 2 years		>2 years	
Regional anesthesia	No^1^; N=43	Yes^1^; N=21	No^1^; N=15	Yes^1^; N=22	No^1^; N=20	Yes^1^; N=19
Age (months)						
Mean (SD)	0.92 (1.20)	2.13 (1.98)	9.8 (3.2)	11.0 (4.6)	107 (59)	69 (52)
Median (IQR)	0.50 (0.30, 1.00)	1.30 (0.40, 3.10)	10.0 (7.9, 10.9)	9.5 (7.6, 13.3)	97 (70, 143)	45 (31, 97)
American Society of Anesthesiologists (ASA)						
4	23 (53.5%)	3 (14.3%)	1 (6.7%)	0 (0%)	0 (0%)	2 (10.5%)
3	20 (46.5%)	16 (76.2%)	12 (80%)	16 (72.7%)	13 (65%)	14 (73.7%)
2	0 (0%)	2 (9.5%)	2 (13.3%)	5 (22.7%)	7 (35%)	3 (15.8%)
1	-	-	0 (0%)	1 (4.6%)	-	-
Surgery						
Coarctation of the aorta	39 (90.7%)	16 (76.2%)	5 (33.3%)	3 (13.6%)	5 (25%)	5 (26.3%)
Vascular ring	4 (9.3%)	5 (23.8%)	10 (67.7%)	19 (86.4%)	15 (75%)	14 (73.7%)
Weight (kg)						
Mean (SD)	3.52 (1.19)	4.78 (1.88)	8.73 (1.68)	9.27 (1.86)	36 (22)	25 (17)
Median (IQR)	3.07 (2.79, 3.93)	4.25 (3.42, 6.11)	8.41 (7.80, 9.41)	8.66 (7.81, 10.41)	24 (22, 51)	17 (16, 24)
Regional method						
Paravertebral block	-	15 (71.4%)	-	13 (59.1%)	-	12 (63.2%)
Erector spinae plane block	-	4 (19%)	-	1 (4.5%)	-	4 (21%)
Caudal block	-	1 (4.8%)	-	8 (36.4%)	-	1 (5.3%)
Serratus anterior plane block	-	1 (4.8%)	-	-	-	2 (10.5%)

Of these 140 patients, 62 (44.3%) received a regional or neuraxial technique along with systemic analgesia, and 78 (55.7%) received only systemic analgesia. All blocks were single-shot blocks. Ages were initially binned into two groups: patients <6 months (N=64; 45.7%) and ≥6 months (N=76; 54.3%). To provide additional rigor, statistical analysis including repeat multivariate logistic regression was utilized again after dividing the patients into three groups, that is, <6 months (N=64; 45.7%), ≥6 months to two years (N=37; 26.4%), and ≥2 years (N=39; 27.9%), to ensure that arbitrary age cutoffs did not change the outcomes/findings of the study. The arbitrary age cutoff to separate >2-year-olds in the repeat analysis served to examine whether an older age group with a perceived improved ability to communicate their state of pain would change any of the measured outcomes. All pain scores documented in the electronic medical record in the first 24 hours post-block were also recorded for each patient, and these pain scores were used to categorize patients into two additional groups based on whether they reported a pain score ≥4 (definition of an episode of moderate pain) at any point during the 24-hour period. Finally, all opioids consumed in the first 24 hours postoperatively were recorded and then converted to intravenous morphine equivalents (IME) using a standard conversion table (see Appendices). 

Primary outcomes

Pain Scores 

Median pain scores by group over time can be found in Table 2. At no time point in the first 24 hours post-block was there a statistically significant difference between pain scores in the two groups.

**Table 2:** Pain scores in the first 24 hours post-block ^1^n (%) ^2^Wilcoxon rank-sum test; Pearson's chi-squared test; Fisher's exact test

Regional	Overall^1^; N=140	No^1^; N=78	Yes^1^; N=62	P-value^2^
Hour 0				
Mean (SD)	1.68 (2.61)	1.39 (2.54)	2.05 (2.68)	0.076
Median (IQR)	0.00 (0.00, 3.00)	0.00 (0.00, 2.00)	0.00 (0.00, 4.75)	
Hour 1				
Mean (SD)	1.30 (2.32)	1.32 (2.43)	1.28 (2.19)	0.84
Median (IQR)	0.00 (0.00, 2.00)	0.00 (0.00, 2.00)	0.00 (0.00, 2.64)	
Hour 2				
Mean (SD)	1.16 (2.02)	1.14 (2.19)	1.19 (1.81)	0.51
Median (IQR)	0.00 (0.00, 2.00)	0.00 (0.00, 1.86)	0.00 (0.00, 2.00)	
Hour 3				
Mean (SD)	1.35 (2.24)	1.21 (2.21)	1.53 (2.29)	0.41
Median (IQR)	0.00 (0.00, 3.00)	0.00 (0.00, 2.00)	0.00 (0.00, 3.00)	
Hour 4				
Mean (SD)	1.40 (2.29)	1.14 (2.15)	1.72 (2.43)	0.1
Median (IQR)	0.00 (0.00, 2.00)	0.00 (0.00, 1.43)	0.00 (0.00, 3.00)	
Hour 5				
Mean (SD)	1.42 (2.22)	1.40 (2.18)	1.44 (2.29)	0.83
Median (IQR)	0.00 (0.00, 3.00)	0.00 (0.00, 3.00)	0.00 (0.00, 2.00)	
Hour 6				
Mean (SD)	1.25 (2.02)	1.27 (2.09)	1.21 (1.94)	0.85
Median (IQR)	0.00 (0.00, 2.00)	0.00 (0.00, 2.00)	0.00 (0.00, 2.00)	
Hour 7				
Mean (SD)	1.02 (1.84)	1.10 (1.95)	0.94 (1.72)	0.63
Median (IQR)	0.00 (0.00, 2.00)	0.00 (0.00, 2.00)	0.00 (0.00, 2.00)	
Hour 8				
Mean (SD)	1.13 (1.96)	0.89 (1.65)	1.43 (2.25)	0.22
Median (IQR)	0.00 (0.00, 2.00)	0.00 (0.00, 1.00)	0.00 (0.00, 3.00)	
Hour 9				
Mean (SD)	1.38 (2.30)	1.47 (2.22)	1.27 (2.41)	0.38
Median (IQR)	0.00 (0.00, 2.21)	0.00 (0.00, 2.86)	0.00 (0.00, 2.00)	
Hour 10				
Mean (SD)	1.14 (1.93)	0.90 (1.77)	1.43 (2.09)	0.12
Median (IQR)	0.00 (0.00, 2.00)	0.00 (0.00, 1.00)	0.00 (0.00, 3.00)	
Hour 11				
Mean (SD)	1.19 (2.01)	0.98 (1.75)	1.44 (2.27)	0.27
Median (IQR)	0.00 (0.00, 2.00)	0.00 (0.00, 2.00)	0.00 (0.00, 3.00)	
Hour 12				
Mean (SD)	1.08 (2.16)	1.06 (2.05)	1.10 (2.30)	0.95
Median (IQR)	0.00 (0.00, 1.71)	0.00 (0.00, 1.57)	0.00 (0.00, 1.50)	
Hour 13				
Mean (SD)	1.14 (2.27)	1.22 (2.48)	1.04 (2.00)	0.9
Median (IQR)	0.00 (0.00, 1.00)	0.00 (0.00, 1.00)	0.00 (0.00, 1.50)	
Hour 14				
Mean (SD)	0.94 (1.85)	0.71 (1.72)	1.23 (1.98)	0.093
Median (IQR)	0.00 (0.00, 0.50)	0.00 (0.00, 0.00)	0.00 (0.00, 2.25)	
Hour 15				
Mean (SD)	0.88 (1.82)	0.85 (1.53)	0.91 (2.16)	0.22
Median (IQR)	0.00 (0.00, 1.00)	0.00 (0.00, 1.43)	0.00 (0.00, 0.00)	
Hour 16				
Mean (SD)	0.88 (1.81)	0.61 (1.52)	1.23 (2.10)	0.12
Median (IQR)	0.00 (0.00, 0.00)	0.00 (0.00, 0.00)	0.00 (0.00, 2.50)	
Hour 17				
Mean (SD)	0.83 (1.88)	1.05 (2.21)	0.57 (1.37)	0.31
Median (IQR)	0.00 (0.00, 0.00)	0.00 (0.00, 1.00)	0.00 (0.00, 0.00)	
Hour 18				
Mean (SD)	1.17 (2.34)	1.14 (2.14)	1.21 (2.57)	0.55
Median (IQR)	0.00 (0.00, 1.21)	0.00 (0.00, 1.86)	0.00 (0.00, 0.00)	
Hour 19				
Mean (SD)	0.98 (2.13)	1.16 (2.14)	0.78 (2.14)	0.26
Median (IQR)	0.00 (0.00, 0.00)	0.00 (0.00, 1.25)	0.00 (0.00, 0.00)	
Hour 20				
Mean (SD)	0.94 (2.00)	1.15 (2.27)	0.70 (1.67)	0.46
Median (IQR)	0.00 (0.00, 0.00)	0.00 (0.00, 0.50)	0.00 (0.00, 0.00)	
Hour 21				
Mean (SD)	0.92 (1.90)	1.11 (1.93)	0.70 (1.88)	0.25
Median (IQR)	0.00 (0.00, 0.00)	0.00 (0.00, 2.00)	0.00 (0.00, 0.00)	
Hour 22				
Mean (SD)	1.41 (2.53)	1.04 (1.97)	1.78 (2.98)	0.6
Median (IQR)	0.00 (0.00, 2.00)	0.00 (0.00, 1.00)	0.00 (0.00, 4.00)	
Hour 23				
Mean (SD)	0.97 (1.90)	0.71 (1.44)	1.19 (2.26)	0.76
Median (IQR)	0.00 (0.00, 1.00)	0.00 (0.00, 0.75)	0.00 (0.00, 1.50)	

**Figure 1:**
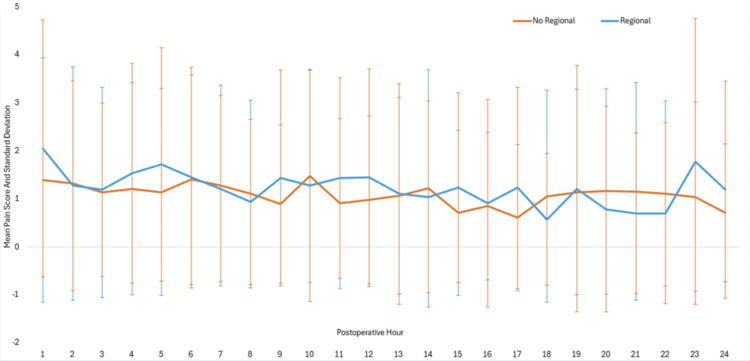
Line graph of mean pain scores (and standard deviation) over time normalized to a 10-point scale in regional and non-regional groups

A graphical representation of mean pain scores over time in regional versus non-regional groups can be found in Figure 1. 

Total Opioid Consumption During the First 24 Hours

The amount of postoperative IV morphine (in mg/kg) consumed in the first 24 hours postoperatively is not associated with either regional anesthesia use (Table 3) or experiencing a max pain score ≥4 (Table 4). The median mg per kilogram IME for the first 24 hours postoperatively was 0.39 mg/kg (0.18, 0.62) in the non-regional group and 0.36 mg/kg (0.20, 0.54) in the regional group (p=0.48). When a subgroup of just paravertebral block patients (the largest regional cohort; N=40) was examined, no difference was found between paravertebral block (0.38 mg/kg (0.23, 0.57)) and the regional group as a whole nor between paravertebral block patients and systemic analgesia alone patients.

**Table 3:** Opioid consumption in first 24 hours postoperatively ^1^n (%) ^2^Wilcoxon rank-sum test; Pearson's chi-squared test; Fisher's exact test

Regional	Overall^1^; N=140	No^1^; N=78	Yes^1^; N=62	P-value^2^
mg of IV morphine equivalents				
Mean (SD)	6 (7)	6 (8)	5 (6)	0.71
Median (IQR)	4 (1, 7)	4 (1, 7)	3 (2, 6)	
Minimum, maximum	0, 36	0, 36	0, 34	
Weight in kg				
Mean (SD)	13 (15)	13 (17)	12 (12)	0.015
Median (IQR)	8 (4, 13)	6 (3, 13)	9 (6, 13)	
Minimum, maximum	2, 96	2, 96	2, 67	
mg/kg of IV morphine equivalents				
Mean (SD)	0.54 (0.69)	0.65 (0.88)	0.39 (0.25)	0.48
Median (IQR)	0.38 (0.19, 0.57)	0.39 (0.18, 0.62)	0.36 (0.20, 0.54)	
Minimum, maximum	0.00, 4.79	0.00, 4.79	0.01, 1.27	

**Table 4:** Multivariate logistic regression: association between age group and maximum pain scores ≥4 after adjusting for confounding factors

Characteristic	OR	95% CI	P-value	Characteristic	OR	95% CI	P-value
Age group				Age group			
<6 months	-	-	-	<6 months	-	-	-
≥6 months	1.9	0.73, 4.87	0.2	6 months to 2 years	1.97	0.65, 6.43	0.2
				>2 years	1.77	0.61, 5.41	0.3
Regional				Regional			
No	-	-	-	No	-	-	-
Yes	0.7	0.32, 1.64	0.4	Yes	0.73	0.32, 1.63	0.4
Surgery				Surgery			
Coarctation of the aorta	-	-	-	Coarctation of the aorta	-	-	-
Vascular ring	2.4	0.89, 6.40	0.085	Vascular ring	2.35	0.89, 6.38	0.086
mg/kg IV morphine equivalents	0.9	0.50, 1.46	0.6	mg/kg IV morphine equivalents	0.86	0.50, 1.46	0.6

Secondary outcomes 

Moderate Pain Incidence 

Ninety-eight patients experienced at least one episode of moderate pain, while 42 did not ever experience a pain score of 4 or greater. Experiencing a pain score of 4 or greater at any time point was not associated with the use of regional anesthesia (Table 5). In univariate models, age group was associated with having experienced moderate pain (≥4), but not in the multivariate model (Table 4). Therefore, there was no statistically significant association between the incidence of moderate pain and age groups (regardless of whether the patients were divided into two or three groups). Overall, 98 (70%) patients (54/78 (69%) of non-regional patients; 44/62 (71%) of regional patients) experienced at least one pain score of 4 or more during their first 24 hours. 

**Table 5:** Secondary outcomes: incidence of moderate pain and time to extubation ^1^n (%) ^2^Wilcoxon rank-sum test; Pearson's chi-squared test; Fisher's exact test

Regional	Overall^1^; N=140	No^1^; N=78	Yes^1^; N=62	P-value^2^
Surgery				
Coarctation of the aorta	73 (52%)	49 (63%)	24 (39%)	0.005
Vascular ring	67 (48%)	29 (37%)	38 (61%)	
Intubated	38 (27%)	31 (40%)	7 (11%)	<0.001
Intubated >24	19 (14%)	18 (23%)	1 (1.6%)	<0.001
At least one episode of pain score ≥4	98 (70%)	54 (69%)	44 (71%)	0.82

Time to Extubation 

There was a statistically significant difference between groups regarding in-OR extubation that favored the regional techniques (31 (40%) non-regional patients remained intubated vs. seven (11%) regional patients; p<0.001), along with those patients who remained intubated >24 hours postoperatively (18 (23%) non-regional patients vs. one (1.6%) regional patients; p<0.001). 

Block-Related Complications 

There were no block-related complications during the five-year study period. Six of the 40 paravertebral blocks performed during the study period utilized clonidine as an adjunct mixed with the local anesthetic (ropivacaine). No adjuncts were added to any of the erector spinae blocks or serratus anterior plane blocks. No adjunct medications were added to the epidural opioid administered in the caudal group (nine exclusively hydromorphone, one exclusively morphine).

## Discussion

In this retrospective case series, we did not find statistically significant differences between regional and systemic analgesia for the many studied outcomes, including postoperative pain scores, opioid consumption on a mg/kg basis, or incidence of moderate pain during the first 24 hours. Both groups demonstrated well-controlled pain, with median and average pain scores <2 in the 24-hour period post-block. Given the well-controlled pain in the systemic analgesia group (with median pain scores <2), it would be very unlikely to observe a statistically significant and meaningful improvement in the regional anesthesia group, especially with this sample size over five years. Thus, a ceiling effect for analgesic improvement may be evidenced in this postsurgical patient population.

These findings, however, do differ from many studies examining regional or neuraxial anesthesia techniques in pediatric cardiac surgical patients who have demonstrated differences in pain control and opioid consumption between regional anesthesia and non-regional anesthesia groups. The efficacy of perioperative analgesia provided by caudal opioid block, erector spinae plane block, serratus anterior plane block, and paravertebral block for thoracotomies or other cardiac surgeries is well-established [7,9,11]. A multitude of randomized trials examining neuraxial techniques in pediatric cardiac surgery in the early 2000s demonstrated lower pain scores [13], reduced opioid consumption [14], shorter time to extubation [15], and a decreased stress response to surgery [2,16]. One group did find no difference in opioid consumption between groups in their prospective randomized trial examining caudal epidural opioids and sham caudal in single ventricle patients undergoing staged palliation [8], which mimics our findings. However, these prior neuraxial patients underwent sternotomy, which involved a cardiopulmonary bypass run, so the findings cannot be directly extrapolated to our thoracotomy patients.

The reason pain scores were reported at hourly intervals (Table 2) was an attempt to capture whether there was a signal that indicates a "block wearing off", which might yield a difference in pain scores between groups at a given time point, as all blocks were single-shot blocks. There was no such time point or difference observed in the first 24 hours, regardless of the regional anesthetic utilized. That outcome prompts further questions, as the caudal epidural morphine or hydromorphone administered in the caudal group should have different pharmacokinetics than any local anesthetic delivered in the paravertebral, erector spinae plane, or serratus anterior plane blocks. This finding gives credence to the thought that multimodal analgesia intraoperatively, in combination with demand-only hydromorphone patient/parent-controlled analgesia pump (PCA) postoperatively (the most common analgesic plan in all-comers), controls pain well enough without the addition of a regional anesthetic that any differences in pain control or opioid consumption are undetectable in a group of this size, namely, that pain is so well-controlled at baseline that regional anesthesia provides no additional, statistically significant relief. Thus, a ceiling effect for analgesic efficacy is possible.

Starting in 2020, the literature describing fascial plane blocks in pediatric cardiac surgery (limited to sternotomies) expanded dramatically. The studies initially examined erector spinae plane blocks and demonstrated decreased opioid consumption [17-19], pain scores [17-19], and time to extubation [17]. Erector spinae plane blocks performed at our institution for the treatment of cardiac surgery by way of thoracotomy did not demonstrate an association with decreased opioid consumption or pain scores when compared to systemic analgesics alone, but they were found to be a part of an analgesic plan that facilitated extubation. Because the study is retrospective, there is undoubtedly selection bias as to which patients were fast-tracked and received regional anesthesia or neuraxial interventions as part of their perioperative care. This was evidenced in the types of patients who received regional techniques. Examples include avoidance of regional in ASA 4 patients (5/29 (17%) ASA 4 patients received regional), more regional offered to older coarctation patients (mean age for regional in the <6-month subgroup was 2.1 months versus 0.92 months), and the majority of vascular ring patients being offered regional blocks when compared to coarctation repairs (24/73 (33%) coarctation patients had regional; 38/67 (57%) vascular ring patients had regional). Thus, the differences found between regional and systemic analgesia groups when it came to in-OR extubation likely reflect the physicians' ability to accurately predict which patients would be fast-track candidates preoperatively, making physician preoperative selection bias a confounding variable to patient extubation time.

After the explosion of interest in erector spinae plane blocks, the predominantly published fascial plane blocks of choice soon transitioned from erector spinae plane blocks to superficial and deep parasternal intercostal plane blocks, which are appealing due to both ease of administration and avoidance of patient repositioning. Thus far, superficial and deep parasternal intercostal plane blocks have been found to decrease opioid consumption, pain scores, and time to extubation [20,21], but are not applicable to thoracotomies due to their parasternal placement and area of dermatomal coverage. However, this body of literature again demonstrates an improvement in cardiac surgical pain metrics with regional anesthesia, which was not observed in our thoracotomy study.

Thus, our finding, namely, that regional anesthetic techniques might not be opioid-sparing nor associated with a change in pain scores when compared to systemic analgesia, does differ from the existing literature. It is not clear whether this finding is unique to thoracotomies, as most of the recent literature examining regional anesthesia in pediatric cardiac surgery examines sternotomy patients only. There are, however, two recent studies that have examined regional anesthesia in coarctation repair thoracotomies. A study by Otu et al. [22] examined regional catheters in coarctation patients and found that when continuous infusions are utilized, there is an associated decrease in opioid consumption. Moreover, a study by Türköz et al. [23] that examined single-shot paravertebral blocks utilized in the management of coarctation repairs in infancy described a lower median IME (0.16 mg/kg in the first 24 hours) when compared to our block patients (0.36 mg/kg), but that study only examined patients who received blocks, so it is unknown what the median morphine consumption was in non-block patients. Ultimately, Otu et al.'s study nicely evidences that these regional techniques can be utilized to reduce opioid consumption when used in a continuous fashion, and Türköz et al.'s study nicely evidences that pediatric thoracotomy patients can be kept comfortable with fairly minimal opioid consumption postoperatively which mirrors our findings.

The nature of the surgical approach to a cardiac lesion, by way of either muscle-sparing thoracotomy or median sternotomy, may affect regional block efficacy. With sternotomy, sustained, wide retraction is necessary most of the time to facilitate bicaval cannulation and the conduct of cardiopulmonary bypass. It follows that translated forces on the chest wall may also yield different pain generators for sternotomy patients than for thoracotomy patients, by way of non-incisional pain. Sternotomy patients may also require multiple thoracostomy tubes, whose locations may fall outside of a block's dermatomal distribution, whereas thoracotomy patients do not always leave the operating theatre with a thoracostomy tube in place at our institution. There are also likely differences in the inflammatory cascade generated from off-bypass thoracotomies when compared to on-bypass sternotomy patients. All of these factors would result in a more comfortable patient postoperatively at baseline, when comparing thoracotomy to sternotomy, which would mean more difficulty demonstrating a difference between standard care and regional techniques.

Although our investigation demonstrated no differences in pain scores or opioid consumption, the routine practice of regional anesthesia in cardiac surgery has persisted at our institution. In many neonates, the intraoperative opioid-sparing nature of pre-incision regional blocks allows for early extubation at procedure end, which would not be possible with an opioid-heavy technique. In older patients, the continued routine use of regional anesthesia can be largely attributed to the request of other peri- and postoperative care providers, including ICU physicians, ICU nurses, and cardiac surgeons. We do recognize, however, that based on our data, we are simply "kicking the can down the road" when it comes to opioid consumption, as patients who had decreased intraoperative opioid administered due to analgesia from a regional technique still received the same mg/kg IME in the first 24 hours as systemic analgesia patients.

Uncontrolled pain in pediatrics has become an unacceptable outcome, with many incentives for prompt and effective treatment. Moreover, local clinical care guidelines and Enhanced Recovery After Surgery (ERAS) protocols have standardized and improved postoperative care. While these patients were not on an ERAS pathway, effective bundles of care are often utilized on patients for whom they were not directly developed, which has consequently raised the standard for clinical care [24]. Such bundles or pathways often change local healthcare provider culture in the process. We attribute the excellent baseline pain control observed in systemic analgesia patients in this and our previous study [9] to clinical care guidelines that have standardized analgesic management in the cardiac ICU. In this milieu of pain control excellence, we can conclude that adjunctive regional techniques are not associated with better pain control when compared to opioid/non-opioid adjuvant-based analgesia alone. 

Our study has certain limitations. First, the retrospective nature of this report limits our ability to determine causality if there had been statistically significant differences observed (which there were not). It also limits sample size, despite having included all of the thoracotomies that occurred for cardiac surgery during the five-year study period. As a result, the study may not be powered to determine the studied effect. Median pain scores were <2 in both groups, suggesting limited room for measurable improvement and a possible ceiling effect in analgesic benefit. Most notably, the regional and non-regional groups exhibited significant baseline imbalance regarding age, weight, ASA physical status, and the specific surgical procedure performed. This heterogeneity introduces a high risk of confounding by indication, particularly concerning our secondary outcome of extubation timing. It is likely that patients who were healthier or undergoing less complex repairs were more frequently selected by the anesthesia team to receive regional blocks as part of a "fast-track" plan. Therefore, the higher rates of in-OR extubation in the regional group likely reflect preoperative clinician selection bias rather than the independent efficacy of the blocks themselves. Despite these variances, no difference in pain or opioid usage was observed. The study's retrospective nature also limits the assessment of any of the blocks' efficacy, which would generally underestimate their efficacy if failed blocks were not appropriately identified in the medical record. Combining several regional anesthetics with local anesthesia and a neuraxial block with opioid (hydromorphone) into a single group (regional anesthesia) for analysis is not ideal, given the heterogeneity and different pharmacokinetics of each medication and block. However, there were simply too few serratus anterior plane blocks (3), erector spinae plane blocks (9), and caudal blocks (10) to examine in isolation. Additionally, given the nature of the retrospective study, the pain scales utilized by bedside nursing staff who were recording pain scores often differed by individual nurse, or by shift, even in the same patient. It is possible that this variability in the pain assessment tools utilized introduced inconsistency in scoring. This discrepancy forced the standardization of pain scores to a 10-point scale, so that scores in an individual could be recorded over time on the "same" scale. Ultimately, utilizing different scales when they weren't standard to a certain patient age or group would have made statistical analysis impossible. Regarding blinding, none existed in this retrospective study. At our heart center, a standardized handoff process is performed involving a report given from the OR team to the ICU team, in which the perioperative analgesic strategy is detailed, which includes whether a regional or neuraxial block was performed during the case. Lastly, the study represents the experience of a single quaternary center where intraoperative anesthetic management was not controlled and, as mentioned previously, selection bias of patients suitable to be fast-tracked with regional anesthetic techniques as part of their care likely occurred. The next step would be a randomized controlled trial with standardized intraoperative management, a blinded regional technique, and standardized postoperative analgesic management.

## Conclusions

In this patient population undergoing thoracotomy for pediatric cardiac surgery, the addition of a regional anesthetic was not found to be associated with decreased pain scores, incidence of moderate pain, or reduced opioid consumption.

## Appendices

**Table 6:** Opioid conversion table for perioperative opioids

To oral morphine equivalents (OME)	
Morphine (IV)	3
Hydromorphone (IV)	21
Fentanyl (IV)	300
Oxycodone (PO)	1.5
Hydrocodone (PO)	1
To intravenous morphine equivalents (IME)	
Morphine (IV)	1
Hydromorphone (IV)	7
Fentanyl (IV)	100
Oxycodone (PO)	0.5
Hydrocodone (PO)	0.33
